# New Appliances

**Published:** 1911-07-22

**Authors:** 


### NEW APPLIANCES.

KELF'S INFANTS' WEIGHT CARD.

(S. Maw, Son and Sons, Aldersgate Street.)

We have received from Mr. H. D. Kelf, chemist, of The Station Pharmacy, Bromley, Kent, a copy of his new infants' weight card, published by Messrs. Maw. Mr. Kelf's patients' weight cards, designed for use at the Southwark Infirmary, are already well known to practitioners and hospital workers; the new addition to the series, in the shape of this infants' weight chart, is likely to prove exceedingly useful, since it is simple, handy, and admirably adapted to its purpose, and enables the variations in weight for the first two weeks to be recorded in ounces.

